# *Medicago ABI3* Splicing Isoforms Regulate the Expression of Different Gene Clusters to Orchestrate Seed Maturation

**DOI:** 10.3390/plants10081710

**Published:** 2021-08-19

**Authors:** David Lalanne, Jaiana Malabarba, Joseph Ly Vu, Michaela Hundertmark, Julien Delahaie, Olivier Leprince, Julia Buitink, Jerome Verdier

**Affiliations:** Institut Agro, INRAE, IRHS, SFR QUASAV, University of Angers, F-49000 Angers, France; david.lalanne@inrae.fr (D.L.); jaiana.malabarba@inrae.fr (J.M.); joseph.ly-vu@inrae.fr (J.L.V.); michaela.bertaud@vilmorin.com (M.H.); julien.delahaie44@gmail.com (J.D.); olivier.leprince@agrocampus-ouest.fr (O.L.); julia.buitink@inrae.fr (J.B.)

**Keywords:** ABI3, seed maturation, desiccation tolerance, *Medicago*, transcriptomics, alternative splicing

## Abstract

Seed maturation comprises important developmental processes, such as seed filling and the acquisition of seed germination capacity, desiccation tolerance, longevity, and dormancy. The molecular regulation of these processes is tightly controlled by the LAFL transcription factors, among which *ABSCISIC ACID INSENSITIVE 3 (ABI3)* was shown to be involved in most of these seed maturation processes. Here, we studied the *ABI3* gene from *Medicago truncatula,* a model legume plant for seed studies. With the transcriptomes of two loss-of-function *Medicago abi3* mutants, we were able to show that many gene classes were impacted by the *abi3* mutation at different stages of early, middle, and late seed maturation. We also discovered three *MtABI3* expression isoforms, which present contrasting expression patterns during seed development. Moreover, by ectopically expressing these isoforms in *Medicago* hairy roots generated from the *abi3* mutant line background, we showed that each isoform regulated specific gene clusters, suggesting divergent molecular functions. Furthermore, we complemented the *Arabidopsis abi3* mutant with each of the three *MtABI3* isoforms and concluded that all isoforms were capable of restoring seed viability and desiccation tolerance phenotypes even if not all isoforms complemented the seed color phenotype. Taken together, our results allow a better understanding of the ABI3 network in *Medicago* during seed development, as well as the discovery of commonly regulated genes from the three *MtABI3* isoforms, which can give us new insights into how desiccation tolerance and seed viability are regulated.

## 1. Introduction

Seeds are the basis of human nutrition and agricultural development. Crop legumes are important food sources for both humans and livestock, ranking third in world crop production, following cereals and oilseeds [1,2]. With approximately 20,000 species, legumes are the third largest angiosperm family, and grain legumes represent 33% of human protein nutrition [3,4]. The understanding of legume seed maturation mechanisms is important for the improvement of legume seed quality traits, and *Medicago truncatula*, a model legume, whose genome has been sequenced and annotated (version 5 [5]), has been very useful to reach this goal in the past decade.

Seed development can be divided into embryogenesis and maturation phases, both implicating a complex web of gene regulation and transcription [6]. During seed maturation, key seed traits are acquired, such as seed germination capacity, seed longevity, dormancy, and desiccation tolerance [7,8]. The acquisition of these processes is essential for seed survival, dispersion, and conquer of dry land environments [9]. In particular, seed desiccation tolerance (DT) provides the necessary protection of the embryo until favorable germination conditions are present [10]. The molecular mechanisms acting on seed maturation are fairly described in model plant *Arabidopsis thaliana.* Seed maturation is mainly controlled by the LAFL regulatory network, which is composed of the genes *LEAFY COTYLEDON1 (LEC1)* and *LEC1-LIKE (L1L)* of the NF-YB gene family, and *ABSCISIC ACID INSENSITIVE3 (ABI3)*, *FUSCA3 (FUS3)*, and *LEC2 (LEAFY COTYLEDON2)* of the B3-AFL gene family [6,11]. The LAFL transcription factors are master regulators of metabolic and developmental pathways because they are capable of triggering a regulatory cascade by activating secondary transcription factors [12]. Genetic and molecular studies unveiled the refined temporal and spatial regulation of the LAFL network, with each gene having a specific function and expression pattern to ensure a proper seed maturation procedure (reviewed by [13]). The B3-AFL proteins comprise up to four conserved protein domains (A, B1, B2, and B3), and the *ABI3* gene is the only one to present all four protein domains [14,15]. Domain A is localized at the N-terminal portion of the protein and is responsible for acidic activation [16]. The B domains vary in size and function. B1 (30 aa) physically interacts with the bZIP transcription factor, e.g., ABI5 (ABSCISIC ACID INSENSITIVE5) [17], while B2 (15 aa) is responsible for the activation of ABA regulated genes through the ABA-response element ABRE [18,19,20], and B3 (100 aa) is a DNA-binding domain [15,21]. The B3 domain is exclusively found in the plant kingdom, making B3 transcription factors plant-specific proteins [22]. From the LAFL network, the *AtABI3* (AT3G24650) gene was shown to be involved in late seed maturation and related to desiccation tolerance, longevity and dormancy, plastid differentiation, and lateral root formation, with its expression positively affected by abscisic acid (ABA) and seed dormancy [23,24,25,26,27,28]. A diverse set of *Arabidopsis abi3* mutants was identified with weak or severe phenotypes, comprising various degrees of ABA insensitivity [14,29,30,31,32,33,34]. The severe *abi3* alleles present high ABA insensitivity, as well as a stay-green embryo phenotype, nondormant seeds, and desiccation intolerance, leading to loss of viability at seed maturity. As the *ABI3* gene is well described and studied in *Arabidopsis*, putative orthologs have been found in many plant species of agronomical interest. In *Medicago truncatula*, *ABI3* (Medtr7g059330/MtrunA17Chr7g0237841) was shown to be specifically expressed on cotyledons after the torpedo stage [35], and its expression was observed from 10 days after pollination (DAP), reaching a plateau at 18 DAP until seed maturity [36]. *Mtabi3* seeds are desiccation-sensitive when dried below a critical water content of 0.4 g DW^−1^, and they also present the stay-green phenotype and ABA insensitivity. Furthermore, *Mtabi3* fully mature and dried seeds completely lose viability and germination capacity [37]. 

Alternative splicing is an important post-transcriptional regulatory system that can increase transcriptome plasticity and proteome diversity [38]. Interestingly, *ABI3* functions might be based on alternative splicing-generated isoforms. *ABI3* expression isoforms have already been identified in dicots such as in *Arabidopsis thaliana* (two isoforms), in *Pisum sativum* (seven isoforms) [39], in *Solanum lycopersicum* (two isoforms) [40], and in *Linum usitatissimum* (three isoforms) [38]; however, specific functional roles of distinct splicing forms are yet to be elucidated. Given that *ABI3* has the capacity of activating a cascade of diverse developmental and metabolic genes for seed maturation, in this study, we identified different expression isoforms of *ABI3* in *M. truncatula.* Then, we describe *MtABI3* expression isoform(s) and evaluate their role to better understand their biological significance for seed maturation processes, including seed survival.

## 2. Results

### 2.1. ABI3 Mutant Transcriptomic Analysis

**Figure 1:**
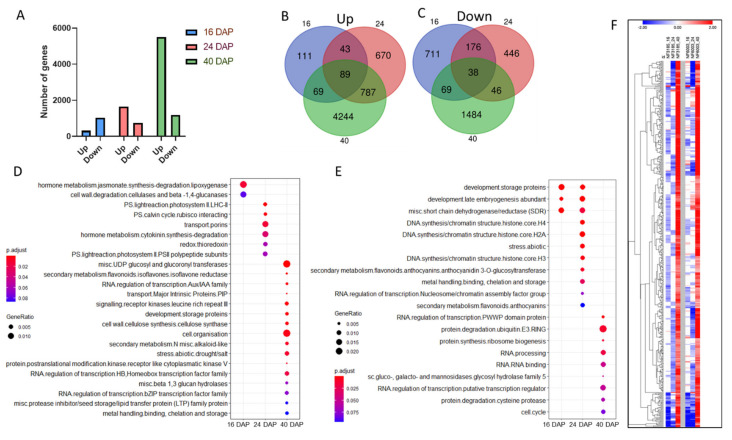
Transcriptomic analysis of *abi3* seeds during seed development. (**A**) Number of differentially expressed genes (DEGs) at each seed developmental stage. (**B**) Venn diagrams of upregulated DEGs at 16, 24, and 40 DAP. (**C**) Venn diagrams of downregulated DEGs at 16, 24, and 40 DAP. (**D**,**E**) Over-representation analysis (ORA) of DEGs in *abi3* at early, middle, and late maturation stage. (**D**) Functional enriched GO terms from upregulated differentially expressed genes. (**E**) Functional enriched GO terms from downregulated differentially expressed genes. The size of the dot represents the gene count. A hypergeometric test was used for statistical analysis, and the *p*-values from the tests were converted to false discovery rate (FDR)-corrected *p*-values as shown in colors, with the red color being more significant than the blue color. (**F**) Heatmap of the 436 genes displaying firstly a downregulation at early seed maturation stages, followed by an upregulation at late maturation stage in both *abi3* mutant lines (NF6003 and NF3185).

To better understand the role of the *ABI3* gene in *Medicago* seeds, we transcriptionally characterized two *Medicago abi3* mutants (NF3185 and NF6003, hereafter referred to as *Mtabi3-1* and *Mtabi3-2* respectively). Both mutants display severe *abi3* phenotypes [37]. These mutants present the chlorophyll retention phenotype, having a green colored mature seed, and they also present a lethal loss of water content during late seed maturation. Furthermore, both mutants are insensitive to ABA; therefore, the application of this phytohormone on mature seeds does not inhibit seed germination [37]. Consequently, these mutants completely lose their viability upon seed maturation. Here, we used *abi3* mutant seeds during seed development to evaluate their transcriptomic state. Our sampling comprised three stages, which characterize early (16 DAP), middle (24 DAP), and late (40 DAP) seed maturation. The *abi3* transcriptomes were compared to a sibling wild-type background at each time point (Supplementary Table S1). Furthermore, we considered as differentially expressed genes (DEGs) only genes that were differentially expressed in both mutants (i.e., either up- or downregulated) at the same stage. We observed DEGs in all three conditions with a higher number of upregulated genes at 40 DAP with more than 5000 genes, when compared with 16 and 24 DAP (312 and 1589, respectively) (Figure 1A). The number of downregulated genes was more homogenous between the maturation stages, with 1041 at 16 DAP, 744 at 24 DAP, and 1176 at 40 DAP. When analyzing the DEGs that were commonly up- or downregulated between the three developmental stages, we observed that *abi3* conserved 89 genes upregulated and 38 genes downregulated when comparing against the wild-type background (Figure 1B). When taking into account the upregulated genes, the common DEGs represented 28% of the upregulated genes at 16 DAP, 5% at 24 DAP, and less than 2% at 40 DAP. This could suggest a more imperative function of these genes at early seed maturation stages. For visualizing DEGs functional roles, we generated a functional enrichment analysis with Mapman gene annotation using Clusterprofiler [41] (Figure 1C,D). We observed that the transcriptomic response for the loss of function of *abi3* was different at each stage of seed development for the upregulated genes (Figure 1C). This difference was also observed on the downregulated genes between 24 and 40 DAP; nevertheless, the downregulated genes at 16 DAP and 24 DAP showed some similar functional classes with seed storage proteins and Late Embryogenesis Abundant (LEA) classes (Figure 1D). Concerning the over-represented functions for each stage on the upregulated genes, we observed at 16 DAP the enrichment of the jasmonate metabolism, while, at 24 DAP, the cytokinin metabolism was enhanced. At 24 DAP, photosynthesis and cellular transport were also enhanced. At 40 DAP, several functional classes showed over-representation with the most significant ones being glucosyl and glucoronyl transferases and cell organization. Moreover, at 40 DAP, RNA regulation of bZIP and homeobox transcription factors were also found over-represented (Figure 1C). When observing the enriched classes of downregulated genes, for early and middle maturation stage, there was an enrichment of development storage proteins and development late embryogenesis abundant (LEA) proteins. At 24 DAP, there was also the enrichment of DNA synthesis with H4 and H2A histones, as well as abiotic stress classes. At the late maturation stage, there was an enrichment of ubiquitin degradation and ribosome synthesis classes. Furthermore, this stage was marked by the downregulation of various RNA regulation classes, such as RNA binding and RNA processing (Figure 1D).

Interestingly, when looking at the list of differentially expressed genes in both *abi3* mutant lines compared to wild-type sibling lines, we identified a set of 436 genes that first displayed a downregulation at early seed developmental stages (i.e., 16 and 24 DAP), followed by an upregulation at seed maturity at 40 DAP (Figure 1E). This specific expression pattern highlighted a set of genes that could be initially directly or indirectly activated by ABI3 at early stages; however, even if they showed an impairment of their regulation at early stages, they displayed activation at later stage using an *ABI3* independent pathway. In this gene set, we identified 17 genes (out of a total of 82 orthologous regulon genes identified in *Medicago*) that were shown to be direct targets of ABI3 in *Arabidopsis* [42] (Supplementary Table S1).

### 2.2. Identification of Three ABI3 Splicing Forms in Medicago Seed Development

**Figure 2:**
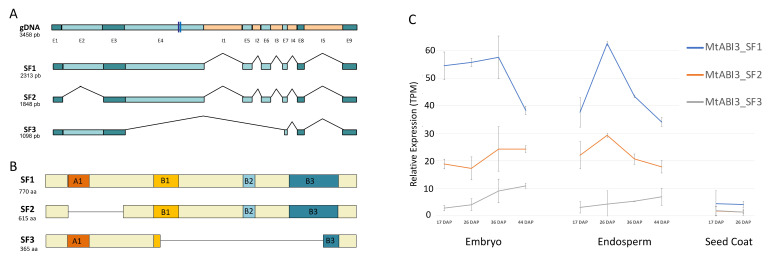
*Medicago ABI3* gene displayed three splicing isoforms. (**A**) Schematic description of the whole gDNA and three splicing isoform structures of *MtABI3* with exons and introns and corresponding size in bp. Dark and blue lines in exon 4 represent the locations of the insertional *Tnt1* transposable elements that characterize the two *Medicago* mutant lines *abi3-1* (NF3185) and *abi3-2* (NF6003). (**B**) Schematic description of different ABI3 protein isoforms with different activating and binding domains and corresponding sizes in amino acids. (**C**) Tentative expression profiling of *ABI3* splicing isoforms using RNA-seq data produced from isolated seed tissues (i.e., embryo, endosperm, and seed coat) at four developmental seed stages using the Salmon algorithm. Relative expressions profiles of isoforms represent the raw read counts normalized by the library size and the transcript length and expressed in transcript per million (TPM).

From a cDNA library screening and sequencing, we identified three different splicing forms for the *MtABI3* gene during seed development encoding three protein isoforms (Figure 2A,B and Supplementary Table S2). From the full-genomic DNA sequence (3458 bp) comprising nine exons and five introns, we identified three transcript forms ranging from 2313 bp to 1098 bp. The longest identified splicing form, called SF1 (2313 bp), contained all nine exons, which potentially encodes a 770 amino-acid protein containing the four functional *ABI3* protein domains with the B1, B2, and B3 interacting domains and the A1 activation domain. The second splicing form, called SF2 (1848 bp), contained eight exons, which encodes a 615 amino-acid protein containing the B1, B2, B3 interacting domains but not containing the A1 activation domain. Lastly, the third splicing form, called SF3 (1098 bp), contained six exons and encodes a short 365 amino-acid protein containing the full length of the A1 activation domain, loss of the B2 domain, and two truncated B1 and B3 domains (Figure 2B). In conclusion, from the identification of the splicing forms, it is to be noted three contrasted gene structures encoding ABI3 proteins with SF1 and SF2 containing the same binding domains with the exception of the A1 activation domain. In contrast, SF3 only contains the full length of the A1 domain and truncated B1 and B3 domains (i.e., 33 amino acids left in the B3 domain, Supplementary Figure S1), which may not be fully functional.

To visualize if these splicing forms could act in different seed tissues or at different seed developmental timing, we analyzed their transcript profiling. After unsuccessful attempts to design suitable and specific primers adapted for qRT-PCR for each of the three splicing forms, we performed in silico transcriptome profiling using the RNA-seq dataset generated from isolated seed tissues at four stages during *Medicago* seed development [43]. This dataset reflects stages at 17, 26, 36, and 44 DAP, which correspond to the onset of seed maturation until seed maturity, when seeds are produced under standard temperature (20 °C day/18 °C night). Early stages, 17 DAP and 26 DAP, were selected as before and after desiccation tolerance acquisition and during the beginning of seed filling and accumulation of storage proteins. Late stages, 36 DAP and 44 DAP, were selected as the onset and after longevity acquisition. Moreover, 36 DAP also corresponds to the end of seed filling and the start of the chlorophyll degradation phase, while 44 DAP represents the pod abscission stage and seed maturity. We used the Salmon algorithm [44], an RNA-seq algorithm able to implement iterations of expectation–maximization to assign reads to specific isoforms and to discriminate between splicing forms. After mapping, read quantification, and normalization to transcript per million (TPM), we predicted that the three *MtABI3* splicing forms (i.e., SF1, SF2, and SF3) displayed expression in the three seed tissues (Figure 2C). Regarding their transcript profiles, the Salmon algorithm estimated that, in embryo, SF1 was highly expressed during seed maturation and finally showed a decrease in expression at seed maturity. In contrast, SF2 and SF3 appeared to display an overall increase of expression from 17 DAP to seed maturity. In endosperm, SF1 and SF2 displayed similar expression with a peak around 26 DAP then a decrease in transcript accumulation until maturity. On the other hand, SF3 in endosperm followed the same trend as in embryo with a constant increase from 17 to 44 DAP. In conclusion, we showed that the *Medicago ABI3* gene was expressed under three splicing isoforms, showing different temporal expression profiles in embryo and endosperm. Moreover, they encoded proteins containing contrasting compositions of activation and binding domains, potentially playing a different role in the seed maturation phase.

### 2.3. Ectopic Expression of the Three Different ABI3 Splicing Forms and Potential Associated Molecular Functions

**Figure 3:**
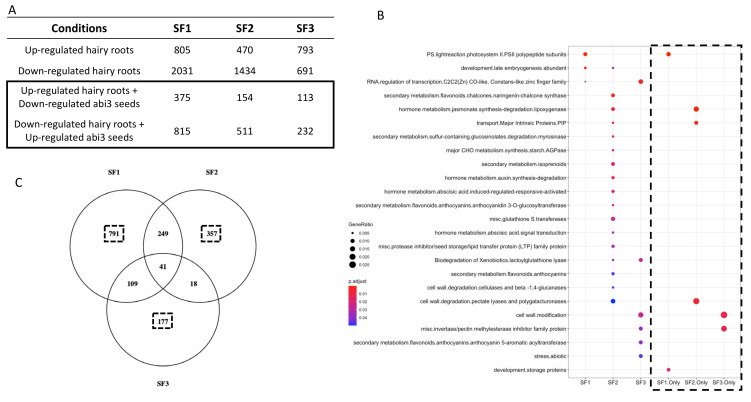
Gene cluster specificity of different splicing forms. (**A**) Number of genes differentially expressed in hairy roots overexpressing the different *ABI3* splicing forms and number of genes potentially regulated by *ABI3* splicing isoforms when combining DEG results from ectopic expression and loss-of-function approaches. (**B**) Functional enriched Mapman terms from gene clusters regulated by SF1, SF2, and SF3 identified from the combination of ectopic expression and loss-of-function approaches. The size of the dot represents the gene count. A hypergeometric test was used for statistical analysis, and the *p*-values from the tests were converted to false discovery rate (FDR)-corrected *p*-values as shown in colors, with the red color being more significant than the blue color. The same analysis (highlighted by a black dashed square) was performed using the genes specifically regulated by each splicing isoform according the Venn diagram (SF1 only, SF2 only, and SF3 only). (**C**) Venn diagrams of different gene clusters regulated by SF1, SF2, and SF3. Numbers with black dashed squares represent the specific gene clusters regulated by each splicing isoform used to perform ORA of SF1 only, SF2 only, and SF3 only.

In order to test if the different splicing forms are involved in specific or overlapping molecular processes, we ectopically expressed the different *ABI3* splicing forms in *Medicago* hairy roots cultivated from the *abi3* loss-of-function mutant lines under the expression of the 35S constitutive promoter. Using *Medicago* hairy roots had many advantages: hairy roots are easily and quickly transformable, they allow ectopic expression of *ABI3* splicing isoforms, and they enable us to observe the isolated effects of each specific splicing form we transformed using hairy roots cultivated from an *abi3* mutant background. After selection of transformed hairy roots, we performed microarray analysis of ectopically expressed splicing forms and identified 4461 and 3604 genes that were, respectively, down- and upregulated by ectopic expression of at least one of the three *MtABI3* splicing isoforms in the *abi3* hairy root system. In more detail, we observed a large proportion of differentially expressed genes when ectopically expressing *ABI3* splicing forms: 2836 DEGs with isoform SF1, 1904 DEGs with SF2, and 1484 DEGs with SF3 (Figure 3A). Interestingly, a high proportion of downregulated genes was observed compared to upregulated genes when ectopically expressing the different splicing forms, which confirmed our previous observation regarding the higher proportion of upregulated genes compared to downregulated genes in the loss-of-function *abi3* mutant lines (Figure 3A). When combining the effect of ectopic expression of splicing forms in hairy roots with the changes in expression in *abi3* mutant lines, we highlighted sets of genes that were either downregulated in mutant lines and upregulated in hairy root ectopic expression or inversely upregulated in mutant lines and downregulated in hairy root ectopic expression, which represent reliable candidate gene sets regulated by different *ABI3* splicing forms. Out of this set of genes, we revealed that a number of them were genes putatively regulated by each isoform: SF1 with 1190 genes, SF2 with 665 genes, and SF3 with 345 genes (Figure 3A). We performed over-representation analysis of gene clusters putatively regulated by their respective splicing forms using ClusterProfiler (Figure 3B). We observed that the gene cluster regulated by SF1 was over-represented by genes mainly related to photosynthesis and photosystem II, as well as late embryogenesis abundant (LEA) protein functional classes. The gene cluster regulated by SF3 was over-represented by genes belonging to “cell-wall modification”, “anthocyanin 5-aromatic acyltransferase”, and “abiotic stress” functional classes. In contrast, we observed many functional classes over-represented in the gene cluster regulated by SF2, such as classes related to hormonal pathways (i.e., jasmonate, auxin, ABA), as well as secondary metabolism with flavonoids, anthocyanins (e.g., chalcone synthase and anthocyanidin 3-*O*-glucosyltranferase), and isoprenoids (Figure 3B).

Lastly, we used a Venn diagram to identify overlapping genes in different gene clusters regulated by different splicing forms and we observed 41 genes common to the three different splicing forms (Supplementary Table S1). Interestingly, most of the SFs appeared to regulate a large proportion of specific genes with 791, 357, and 177 genes specifically regulated by SF1, SF2, and SF3, respectively (Figure 3C). According to this result showing a relative gene specificity of gene clusters from the different splicing forms, we refined our over-representation analysis to obtain a more stringent vision of functional classes specifically regulated by different splicing forms (Figure 3B). We confirmed an over-representation of genes involved in the photosynthesis and photosystem II and “development storage protein” classes regulated by SF1. Specific genes regulated by SF2 showed over-representation of genes related to lipoxygenase, plasma membrane intrinsic protein (PIP), and cell-wall degradation enzymes. Finally, SF3 specific genes showed an over-representation of genes involved in cell-wall modification and pectin methyltransferases (Figure 3B). In conclusion, we observed a relatively high specificity in gene clusters regulated by different *ABI3* splicing isoforms with few commonly regulated genes (i.e., only 41 regulated by the three splicing forms). Moreover, these specific gene clusters were involved in contrasting molecular functions, suggesting a specific molecular role of each splicing form during seed development.

### 2.4. Complementation of Arabidopsis ABI3 Mutant Lines Using the Three Medicago Transcript Isoforms

**Figure 4:**
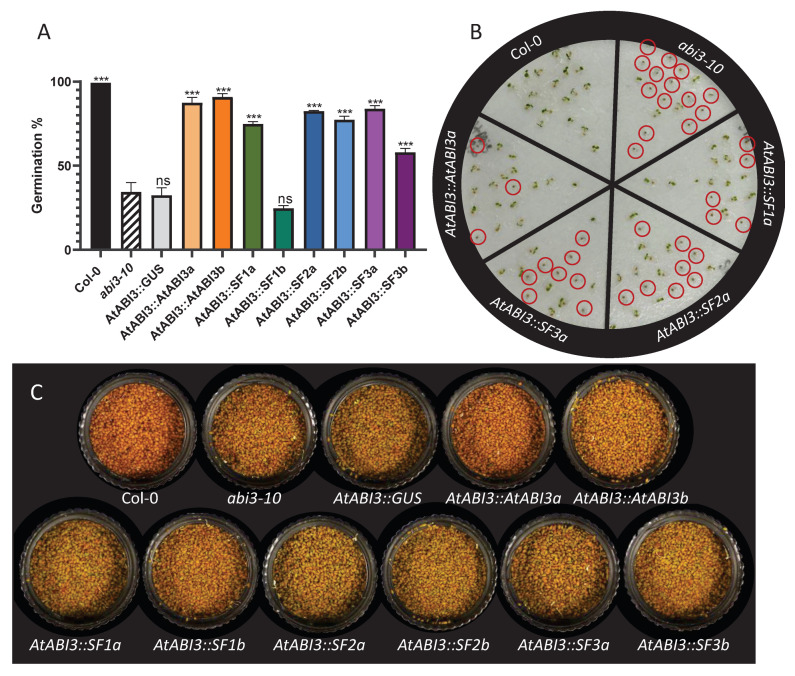
Complementation of *Arabidopsis abi3-10* mutant with *M. truncatula ABI3* isoforms. (**A**) Germination percentage of mature seeds from wild-type background (Col-0), *Atabi3-10* mutant, empty vector (*AtABI3::GUS*), and complemented lines with *Arabidopsis* full-length transcript (*AtABI3::AtABI3*). Three technical replicates of 100 seeds each were used. One-way ANOVA showed that all genotypes were significantly different from the *Arabidopsis abi3* mutant, with the exception of the empty plasmid (*AtABI3::GUS*) and line containing *AtABI3::SF1b*. ‘ns‘ indicates a nonsignificant difference and ***: *p* ≤ 0.001. (**B**) Illustration of germination test showing Col-0, *abi3-10*, *AtABI3* complementation, and *M. truncatula ABI3* isoform complementation. Red circles indicate non-germinated seeds (**C**) Mature and dried seed colors. Controls genotypes are shown in the upper panel, and *M. truncatula ABI3* isoform complemented lines are shown in the lower panel.

To elucidate if different *Medicago* splicing forms were functional, we performed a complementation experiment of *Arabidopsis abi3-10* mutant lines using *Medicago* SF1, SF2, and SF3 sequences under the expression of the native *ABI3* promoter of *Arabidopsis*. Mature seeds from two independent transformed lines (named a and b) were first dried, and then dormancy was released before performing a germination assay for each splicing form and corresponding controls (Figure 4A,B). First, we observed that the initial *abi3-10* mutant lines and the *abi3-10* complemented line with the *GUS* gene (*AtABI3::GUS*) displayed a low germination rate, about 30%, confirming the effect of *abi3* mutation on the survival rate of mature seeds, mainly due to desiccation intolerance. Complementation with the *Arabidopsis* full-length transcript sequence (*AtABI3::AtABI3*) almost restored the seed germination phenotype close to the wild-type germination rate (Col-0). Interestingly, we also observed that complementation with any of the *Medicago* splicing forms (i.e., *AtABI3::MtSF1*, *AtABI3::MtSF2*, and *AtABI3::MtSF2)* largely restored the seed germination and seed survival rates of the *abi3-10* mutants, except for the second complementation lines with SF1b that did not show a complementation phenotype (Figure 4A). To illustrate these germination percentage results obtained on triplicates of 100 seeds for each construct, we performed a germination assay on a lower number of seeds and visualized similar results with germinated seeds compared to non-germinated seeds, indicated in red circles (Figure 4B). On the other hand, the *ABI3* gene is also known to be involved in seed degreening during maturation; thus, we looked at the complementation of this phenotype in our different complemented lines. We observed the characteristic green color of mature seeds from *abi3-10* mutant lines, as well as the empty vector line containing the *GUS* gene, compared to the brown color of Col-0 and *AtABI3::AtABI3a* mature seeds. However, our second complemented line containing the *AtABI3::AtABI3b* construct did not show proper complementation of seed degreening and still appeared green. This was also seen for the *Medicago* splicing isoforms that did not complement the seed degreening phenotype of *abi3-10* mutants. However, these results on complementation of seed germination/viability and degreening showed that we can uncouple these two mechanisms.

## 3. Discussion

### 3.1. Alternative Splicing of ABI3 during Medicago Truncatula Seed Development

Alternative splicing is an important post-transcriptional mechanism, which has been shown to be implicated in many plant responses to environment and regulation of developmental processes (as reviewed in [45]). Indeed, the possibility of a gene to present alternative splicing of its mRNA can promote gene advantage due to this important post-transcriptional regulatory mechanism, since it can impact mRNA stability and, at the same, time increase protein diversity. The proportion of genes undergoing alternative splicing has largely been underestimated; using RNA and genomic sequencing technologies, it appeared that between 20% and 60% of genes containing multiple exons could generate more than one splicing isoform depending on plant species and environmental conditions [46,47]. Alternative splicing events have been shown to be highly active during embryogenesis and seed maturation. As an example, in soybean during embryogenesis, it has been shown that 47,331 genes were able to generate 217,371 different transcript isoforms [48]. As another example, in *Arabidopsis*, 34% of genes expressed during desiccation tolerance acquisition during seed maturation were shown to be alternatively spliced, suggesting a link between intense alternative splicing activity and ABA-related factors [49]. In this present study, we detected the presence of three splicing isoforms of *MtABI*3 expressed during seed maturation called SF1, SF2, and SF3, even if additional *MtABI3* splicing isoforms might occur at different developmental stages or stress conditions. By stating that isoform SF1 is the complete protein, since it comprises all the domains (A1, B1, B2, and B3), isoforms S2 and S3 are characterized as an exon-skipping alternative splicing, with the loss of exon 2 for SF2 and the loss of multiple exons (4, 5, and 6) for SF3. Exon skipping is a relatively rare event and represents 8% of total alternative splicing events in *A. thaliana*, affecting less than 2% of its genes. In contrast, intron retention alternative splicing represents 56% of the splicing events and occurs in almost 15% of genes in *A. thaliana*. Not surprisingly, other *ABI3* genes were reported to display varying splicing isoforms. For instance, in dicots, two isoforms were observed in *Arabidopsis* and tomato [40,50], while seven isoforms have been reported in pea [39]. Similarly to our study, *ABI3* isoforms from *Arabidopsis*, tomato, and pea are temporally regulated during seed development (Figure 2C). The role of splicing isoforms is still unclear even if many different hypotheses exist. For instance, regarding *PIF6,* long splicing isoforms were shown to be expressed in response to light in seedlings, while the short isoforms are only expressed during germination [51]. On the other hand, the four splicing isoforms of *DOG1* were shown to be independently functional to regulate seed dormancy, but the presence of several isoforms is required for complete gene function by preventing protein degradation [52]. Many other hypotheses regarding the role of splicing isoforms have been elaborated such as DNA- and protein-binding strengths, which could differ according to presence or absence of binding domains in different protein isoforms, such as *ABI3* or *ABI5* [39,40].

### 3.2. Splicing Isoforms of Medicago ABI3 Regulate Different Set of Genes but Restore Seed Viability Phenotype of Mutant Lines

As described in many species, ABI3 has a pleiotropic effect on seed maturation processes including seed filling, desiccation tolerance, dormancy, longevity, and loss of chlorophyll content. In our study, we showed that, in the loss-of-function mutants, many molecular functional classes and processes were transcriptionally impaired such as storage protein, LEA, secondary metabolism, and cell cycle-related genes throughout seed maturation (Figure 1D). Interestingly, we found that 436 genes were first downregulated in the *abi3* mutant lines at early stages of seed maturation and then eventually upregulated at the late maturation stage (i.e., 40 DAP) (Figure 1E). Some of these genes (Supplementary Table S1) are known to be either indirectly (e.g., *ABI5*) [53] or directly (e.g., *LEA4-5, Oleosin1*) regulated upstream of *ABI3* [42]. Their delayed activation in the *abi3* mutants suggests the existence of an *ABI3* independent pathway to activate the expression of these genes. As a hypothesis regarding the delayed activation of *ABI5* in *abi3* mutant, several studies showed that ABI3 and ABI4 were positive regulators of *ABI5* expression during seed germination and seedling development [23,53,54], but we cannot verify if this independent ABI3 pathway is linked to ABI4 as we do not have its expression profile, which is not included in the Nimblegen Medtr v1.0 12 × 135K arrays. Interestingly, we also noticed in this study a large set of genes upregulated in the later maturation stage in the loss-of-function *abi3* mutants and, in contrast, a large proportion of genes downregulated by ectopic expression of different splicing forms in the *abi3 Medicago* hairy roots (Figure 1A and Figure 3A), suggesting a direct or indirect repressor effect of this gene. Indeed, some B3 domain proteins expressed during seed maturation, including the ABI3/VP1 family, have been shown to contain putative and novel active repression domains in *Arabidopsis* [55], which could explain the ability of ABI3 to directly or indirectly repress gene expression.

The presence of multiple splicing isoforms of *MtABI3* during seed development suggests a possible role of the alternative splicing isoforms in biological functions. Our study aimed to investigate the role(s) of *ABI3* isoforms of *Medicago truncatula* in two heterologous models, firstly using ectopic expression of *MtABI3* splicing forms in *Medicago* hairy roots impaired in native *abi3* function and secondly using *Arabidopsis abi3* complementation using *Medicago* ABI3 isoforms, to conclude about the impact of each alternative ABI3 protein on the regulation of downstream genes involved in seed maturation processes and seed viability. Using ectopic expression in *Medicago* hairy roots, our results showed that each isoform was responsible for the activation of a specific set of genes, along with a small number of commonly regulated genes. Functional annotation and over-representation studies of these unique sets of genes showed that they could regulate specific and distinct molecular processes, suggesting a specific role of different splicing isoforms. This hypothesis is coherent with the temporal differences in splicing isoform expression profiles and with the contrasted composition of protein domains between different splicing isoforms with SF1 containing all A1, B1, B2, and B3 conserved domains, whereas A1 is absent in SF2 and only A1 and truncated B1, B3 are present in SF3 (Figure 2B). Some studies described the role of these domains, such as the B1 domain being essential for ABI5 interaction [17] and the B3 domain being essential in DNA binding to interact with RY elements [15] and indirectly to G-boxes via bZIP [17,56]. Regarding SF3 containing the A1 and truncated B1 and B3 domain, it could act as the short *Arabidopsis* splicing form *ABI3*-b transcript containing the A1 acidic transcriptional activation domain and the first binding domain, which can still mediate ABA signaling during late seed maturation [50], or it could act as the PsABI3-5 isoform from pea, which was shown to be functional and contain only the A1 domain with a portion of the C-terminal extension closely related to it [39]. The existence of a deleted or a truncated domain in SF2 and SF3 could also reduce protein/DNA-binding strength and could compete with other splicing forms containing the complete domains to modulate ABI3 functions, as observed with the repressing VAL proteins [57]. This hypothesis of reduced binding capacity of SF3 is strengthened by a recent work regarding the structural characterization of the B3 domain of ABI3 gene, which showed that the ABI3-B3 domain recognizes a consensus DNA sequence called Sph/RY (5′–TGCATG–3′) via a conserved set of base-specific contacts [58]. The amino acids involved in the B3 domain/DNA binding form a protein structure called the N-arm and C-arm, due to its binding orientation. The N-arm of the AtABI3 protein is composed of amino acids at positions 576 to 591 and the C-arm is located at positions 622 to 639, both inside the B3 domain (Supplementary Figure S1). The SF3 isoform of *M. truncatula* completely loses the N-arm, as well as almost the totality of the C-arm (i.e., two amino acids left), which suggests a decrease in B3 binding capacity for this isoform. Even if we observed activation of different sets of genes for each splicing form, when performing the complementation experiment of *Arabidopsis abi3* mutant using *Medicago* splicing forms, we observed a similar restoration of seed survival phenotype; thus, we cannot exclude that SFs also share redundant functions in seed desiccation tolerance for instance. Indeed, *abi3-10* mutant lines displayed a low survival rate at seed maturity marked by a low germination rate. Concerning the phenotypes of *Arabidopsis abi3-10* complemented mutants, all the three isoforms of *M. truncatula ABI3* were sufficient to restore most of seed viability and, therefore, seed desiccation tolerance, during seed maturation (Figure 4A). These results showed that domains A1 and B2 are not essential for seed survival and acquisition of desiccation tolerance, since isoforms SF2 (lack of A1 domain) and SF3 (lack of B2 domain) can still achieve seed germination with A1 and truncated B1 and B3 domains. Studies with the B3 domain deficiency due to *abi3-12* mutation indicate that ABI3 activation of the LAFL network requires the B3 DNA-binding domain [59]. This could suggest that the C-terminal region of the B3 domain of MtABI3 still present on SF3 after exon skipping alternative splicing is sufficient to perform DNA binding to target genes and to initiate the diverse mechanisms required to ensure seed survival. From this perspective, the 41 putative target genes shared by the three splicing forms could be important players in seed survival, even if this gene set list might not be exhaustive due to the presence of about 55% of *Medicago* genes on the genechip used in this study (23,536 genes on gene chip out of 42,875 genes identified from *Medicago* Genome version 5, [5]). Lastly, these *Arabidopsis* lines showing nearly complete complementation of seed survival phenotype, even with the minimal domain composition of *Medicago* isoform SF3, represent an important tool to continue to deepen our knowledge about *ABI3* gene function and its pleiotropic effect on seed maturation and survival.

## 4. Materials and Methods

### 4.1. Plant Material

*Medicago truncatula* homozygous *Tnt-1* insertion mutants *abi3-1* (in R108 genetic background, NF3185) and *abi3-2* (in R108 genetic background, NF6003) were obtained from the Noble Research Institute (Ardmore, OK, USA). Homozygous seeds were produced and isolated from a previous study (Delahaie et al., 2013). Seeds were germinated in round (Ø8 cm) square pots with Green Fibre substrate 5 (ref.666, Klasmann-Deilmann, Bourgoin Jallieu, France) and transferred after genotyping to 2 L round pots containing the same substrate. Growth chamber conditions were set to an average temperature of 19.5 °C (20 °C/19 °C day/night) with a 16 h light/8 h dark photoperiod and a light intensity of 150 μE·m^−2^·s^−1^ and controlled humidity (60–70%).

*Arabidopsis thaliana* Col-0 plants and T-DNA insertion mutant *abi3-10* (in Col-0 background, SALK 023411) were grown in (Ø8 cm) pots with Tray substrate 75/25 (ref.092, Klasmann-Deilmann, Bourgoin Jallieu, France). Growth chamber conditions were set to an average temperature of 20 °C (22 °C/18 °C day/night) with a 16 h light/8 h dark photoperiod with a light intensity of 150 μE.m^−2^.s^−1^ and controlled humidity (60–70%).

### 4.2. RNA Extraction and Hybridization Arrays

For the loss-of-function study, seeds of *Medicago truncatula abi3-1* (NF6003), *abi3-2* (NF3185), and corresponding sibling wild-type lines R108 were freshly harvested from three different developmental stages (16, 24, and 40 days after pollination (DAP)) and directly frozen in liquid nitrogen. Total RNA was purified using the NucleoSpin^®^ RNA Plus extraction kit (Macherey-Nagel, Düren, Germany), following the manufacturer’s instructions. Quantity and quality of RNA were measured using a NanoDrop ND-1000 (NanoDrop Technologies, Thermo Fisher Scientific, Waltham, MA, USA), and integrity was checked using a Bioanalyzer Agilent 2100. RNA amplification, labeling, and hybridization of Nimblegen Medtr v1.0 12 × 135K arrays were performed according to Terrasson et al., 2013 [60]. Three biological replicates were analyzed per comparison using the dye-swap method, and statistical analysis on the gene expression data was performed according to Terrasson et al., 2013 [60]. Raw and analyzed transcriptome data are openly available on the Gene Expression Omnibus platform as GSE181013 and GSE180917.

### 4.3. Cloning and Ectopic Expression in Hairy Root

Ectopic overexpression of *MtABI3* splicing forms, called SF1 (2313 bp), SF2 (1848 bp), and SF3 (1098 bp), was performed on *Medicago* hairy roots generated from *Medicago* roots of the *abi3* mutant background. For this aim, the different amplicons of each splicing form were amplified with CACC tag motif before the ATG codon and inserted into the pENTR™/D-TOPO^®^ vector (Invitrogen, Waltham, MA, USA) following manufacturer’s instructions. These constructs were then cloned into OneShot^®^ TOP10 *E. coli* (Invitrogen) and transferred into the pK7WG2D,1 vector [61] using the Gateway^®^ LR Clonase^TM^ II Enzyme Mix recombination system (Invitrogen). pK7GW2D,1 contains a 35S cauliflower mosaic virus promoter and a GFP reporter that was used as a transformation marker, as reported in Verdier et al., 2012 [62]. As an empty vector control, we cloned the *GUS* gene into the same plasmid that was used to normalize our results (i.e., *35S::SF1-GFP/35S::GUS-GFP*, *35S::SF2-GFP/35S::GUS-GFP, 35S::SF3-GFP/35S::GUS-GFP*). Final constructs were transformed into *Agrobacterium rhizogenes* strain ARqua1 by electroporation [63]. Then, following the protocol from Chabaud et al., 2003 [64], transformed colonies were grown on LB agar medium at 28 °C, with spectinomycin and streptromycin for vector selection. After confirmation by PCR, transformed *Agrobacteria* were used to transform 3 mm germinated radicles of *Medicago truncatula abi3-2* mutant (NF6003). The resulting hairy roots were maintained on Fahraeus agar medium in Petri dishes supplied with 25 mg·L^−1^ kanamycin under fluorescent light (150 μE·m^−2^·s^−2^) with a 16 h photoperiod. Screening of transformed hairy roots was performed using GFP fluorescence under UV microscope (Olympus U-RFL-T, Tokyo, Japan) and transformed hairy roots were harvested in liquid nitrogen. RNA was extracted from three replicates of similar amounts of roots using Nucleospin RNA plus Purification Kit (Macherey Nagel, Düren, Germany).

### 4.4. Arabidopsis ABI3 Complementation with Different Medicago Splicing Forms

In order to evaluate the capacity of *MtABI3* isoforms to complement the *Arabidopsis abi3* phenotype, *abi3-10* mutant lines (N659724, SALK_023411) were transformed with vectors containing the 3 kb *Arabidopsis ABI3* promoter fused with the coding sequence (CDS) of *Medicago* splicing isoforms. The same pENTR™/D-TOPO^®^ vectors containing the three splicing isoforms of *MtABI3*, described previously, were initially used to transfer the different constructs into the destination vector pKWG using Gateway^®^ LR Clonase™ II Enzyme Mix recombination system (Invitrogen) leading to four constructs: *AtABI3::SF1, AtABI3::SF2, AtABI3::SF3*, and the control construct containing *AtABI3::AtABI3*. Moreover, a pKWG vector containing the CDS of the GUS gene was used as a empty vector control (*AtABI3::GUS*). These constructs were transformed into thermo-competent *Agrobacterium tumefaciens* strain AGL1. The floral dip method [65] was used to transform *abi3.10* plants, and T1 seeds were initially germinated in MS culture medium (Sigma, St. Louis, MO, USA) and selected for kanamycin resistance (75 μg·mL^−1^). Two-week-old putative transformants were screened by PCR using specific primers targeting the plasmid promoter and the *MtABI3* isoform coding regions. A second generation (T2) was generated, and seeds from T2 (i.e., T3 plantlets) were selected using antibiotics to isolate T2 homozygous plants used for the phenotyping assays.

Seed germination assays were performed on two independent complemented *Arabidopsis* lines with triplicates of 100 seeds. Mature seeds were first dried for 3 days under an airflow at 44% relative humidity (RH), and then stratified for 72 h at 4 °C, followed by imbibition on wet filter paper at 20 °C, 16 h light. Seed germination was observed for 10 days, with observations every 3 days. The final rate of germination was scored after 10 days of imbibition.

### 4.5. Expression Profiles of MtABI3 Splicing Isoforms Using Existing RNA-Seq Data

To identify the expression profiles of the three *Medicago* splicing isoforms, we used RNA-seq data generated previously [43] (GSE160725) representing transcriptomic profiling of four developmental seed stages in the three *Medicago* seed tissues. High-quality reads were mapped against *Medicago* reference transcriptome version 5 (release 1.7, https://medicago.toulouse.inra.fr/MtrunA17r5.0-ANR/), where the three different *ABI3* splicing isoforms were manually added to the reference transcriptome, using a quasi-mapping alignment, and they were quantified using the Salmon algorithm v.1.2 (https://combine-lab.github.io/salmon/ and accessed on 2020), one of the most robust mappers/quantifiers to discriminate between multiple transcript isoforms [44,66]. For gene expression analysis, raw RNA-Seq data were first normalized as transcripts per million (TPM) using transcript lengths and library sizes.

### 4.6. Gene Annotation and Over-Representation Analysis

Gene annotation of *Medicago truncatula* transcripts version 5 (release 1.7) was performed using Mercator4 (version 3, https://www.plabipd.de/portal/mercator4 (accessed on 8 August 2021)), and transcripts were annotated using the MapMan plant functional classification [67]. Over-representation analyses (ORA) of MapMan bin were performed using the ClusterProfiler package [41] in RStudio (version 1.3.1073, https://bioconductor.org/packages/release/bioc/html/clusterProfiler.html and accessed on 8 August 2021) applying an adjusted *p*-value cutoff of <0.05 obtained after a Bonferroni–Hochberg procedure.

## Data Availability

Raw and analyzed microarray data presented in this study are openly available on the NCBI website using the accession numbers GSE181013 (*abi3* loss-of-function mutant analysis) and GSE180917 (ectopic expression analysis of *Medicago* splicing isoforms in hairy roots) or directly at https://www.ncbi.nlm.nih.gov/geo/query/acc.cgi?acc=GSE180917 (accessed on 8 August 2021) and https://www.ncbi.nlm.nih.gov/geo/query/acc.cgi?acc=GSE181013 (accessed on 8 August 2021).

## Supplementary Materials

The following are available online at www.mdpi.com/article/10.3390/plants10081710/s1: Supplementary Table S1. Summary of transcriptomic data generated in this study. Log_2_ ratio normalized against sibling wild-type lines for loss-of-function mutant analysis (NF3185 and NF6003) (in green) and against hairy root transformed with GUS construct for ectopic expression of different splicing forms in *Medicago abi3* hairy roots (in blue). Information about gene putative functions, tentative annotations, and Mapman functional annotations is indicated in gray. We also indicated genes displaying the down- then upregulation expression pattern as discussed in the manuscript and the putative ABI3 regulons identified in [42]; Supplementary Table S2. Nucleic and amino-acid sequences of the three *Medicago* splicing isoforms with respective size in base pairs or amino acids; Supplementary Figure S1. N-arm and C-arm of the B3 domain from *MtABI3* isoforms and *AtABI3*.
