# Supplementary material for: Medicago ABI3 Splicing Isoforms Regulate the Expression of Different Gene Clusters to Orchestrate Seed Maturation
Source: Plants (Basel). 2021 Aug 19;10(8):1710. doi: 10.3390/plants10081710 (PMC8398556; doi:10.3390/plants10081710)
Supplement: Supplementary file 1 [file Supplementary Figure S1.pdf › Supplementary Figure S1.pdf]

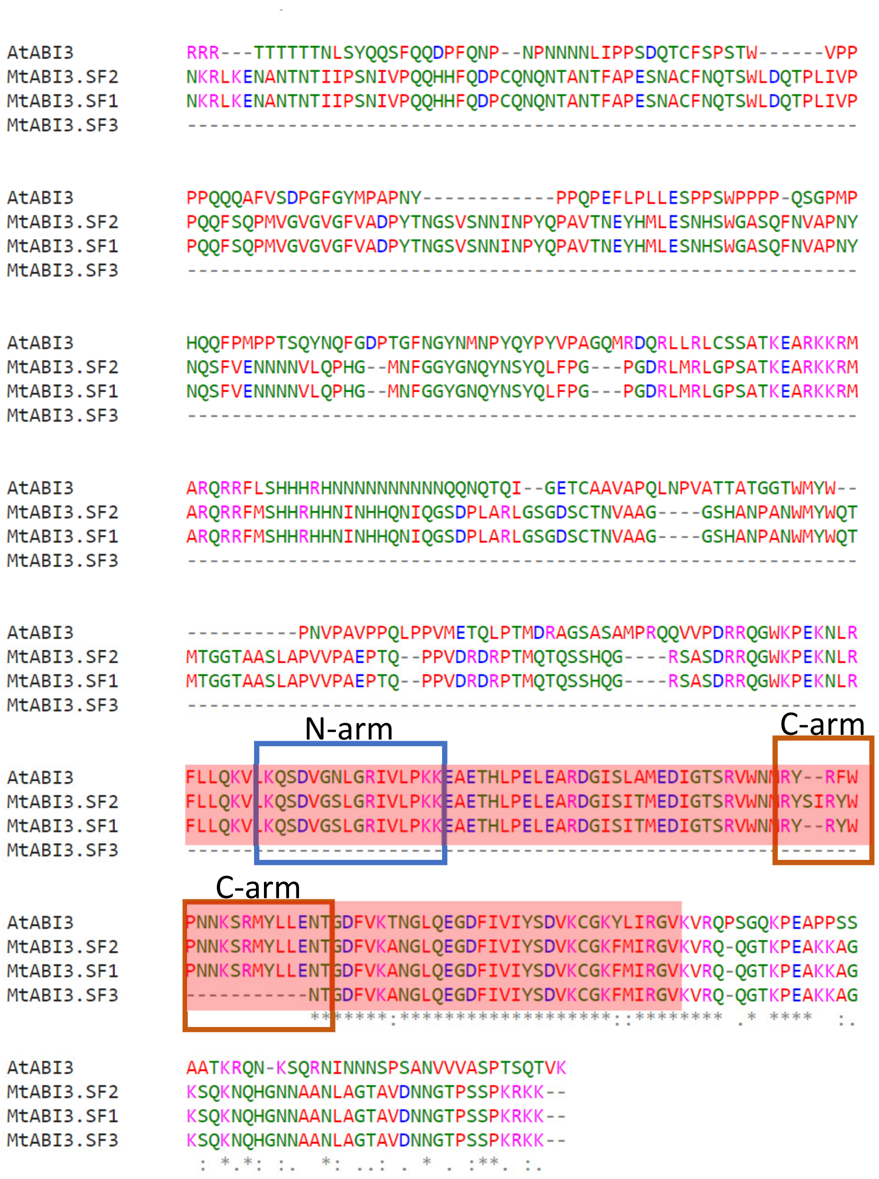

Figure S1: N-arm and C-arm of the B3 domain from *MtABI3* isoforms and *AtABI3*. B3 domain is colored in red.

B3 Domain
